# Supplementary material for: Physical function domain associations with cognitive domains in community-dwelling older adults
Source: medRxiv. 2026 Jul 1:2026.06.29.26356840. Preprint. [Version 1] doi: 10.64898/2026.06.29.26356840 (PMC13345577; doi:10.64898/2026.06.29.26356840)
Supplement: 1 [file NIHPP2026.06.29.26356840V1-supplement-1.pdf]

Supplementary Tables:

Supplementary Table 1. Influence of Potential Covariates on Cognitive Measures Excluding Males

	MoCA	LSWM*	PSM*	Flanker*	DCCS*
Model (*F(4,44)*, R^2, *Adj.* R^2, *p)*	1.55, 0.12, 0.04, 0.203	1.58, 0.13, 0.05 0.198	2.42, 0.18, 0.11, 0.063	0.81, 0.07, -0.02, 0.523	1.39, 0.11, 0.03, 0.252
Age (β, *p*)	-0.14, 0.335	-0.04, 0.786	**-0.38, 0.010**	-0.10, 0.494	-0.26, 0.083
Education (β, *p*)	0.22, 0.139	**0.31, 0.041**	0.15, 0.292	-0.15, 0.320	-0.14, 0.337
BMI (β, *p*)	0.18, 0.233	-0.05, 0.728	0.08, 0.284	-0.16, 0.290	-0.11, 0.456
PP (β, *p*)	0.19, 0.193	0.14, 0.345	-0.04, 0.755	0.20, 0.190	-0.10, 0.511

Note: * - indicates that values are uncorrected standardized scores; Adj. R^2 – Adjusted R^2; BMI – body mass index; PP – pulse pressure; MoCA – Montreal Cognitive Assessment; LSWM – List Sort Working Memory; PSM – Picture Sequence Memory; DCCS – Dimensional Change Card Sort

Supplementary Table 2. Summary of Regression Analyses Examining the Association Between Physical Function and Cognition Excluding Males

	MoCA	LSWM*	PSM*	Flanker*	DCCS*
Model ($\Delta F(1,43)$, ΔR^2)	0.26, 0.01	0.75, 0.02	0.17, 0.00	0.13, 0.00	0.77, 0.02
Handgrip† (B [95% CI])	-0.04 [-0.19, 0.11]	-0.25 [-0.83, 0.33]	0.16 [-0.63, 0.95]	0.10 [-0.44, 0.64]	0.25 [-0.32, 0.82]
Handgrip†(β, *p*)	-0.08, 0.611	-0.13, 0.393	0.06, 0.681	0.06, 0.718	0.13, 0.384
Model ($\Delta F(1,43)$, ΔR^2)	0.54, 0.01	0.48, 0.01	0.31, 0.01	0.51, 0.01	0.09, 0.00
30s Chair Stand (B [95% CI])	0.08 [-0.15, 0.31]	0.30 [-0.58, 1.18]	0.33 [-0.86, 1.52]	0.29 [-0.52, 1.10]	0.13 [-0.74, 1.00]
30s Chair Stand (β, *p*)	0.12, 0.466	0.11, 0.494	0.09, 0.579	0.12, 0.477	0.05, 0.769
Model ($\Delta F(1,43)$, ΔR^2)	3.21, 0.06	5.85, 0.11	0.17, 0.00	2.10, 0.04	1.55, 0.03
TUG† (B [95% CI])	-0.42 [-0.89, 0.05]	-2.10 [-3.86, -0.35]	-0.51 [-3.03, 2.01]	-1.22 [-2.91, 0.47]	-1.12 [-2.93, 0.70]
TUG† (β, *p*)	-0.32, 0.080	-0.42, 0.020	-0.07, 0.685	-0.27, 0.155	-0.23, 0.221
Model ($\Delta F(1,43)$, ΔR^2)	4.12, 0.08	1.17, 0.02	4.70, 0.08	2.77, 0.06	1.21, 0.02
10-Meter Walk† (B [95% CI])	-0.99 [-1.98, -0.01]	-2.10 [-6.01, 1.81]	-5.57 [-10.56, -0.38]	-2.94 [-6.50, 0.62]	-2.10 [-5.96, 1.75]
10-Meter Walk† (β, *p*)	-0.34, 0.049	-0.19, 0.285	-0.35, 0.036	-0.29, 0.104	-0.19, 0.277
Model ($\Delta F(1,43)$, ΔR^2)	8.59, 0.15	1.49, 0.03	**18.42, 0.25**	1.92, 0.04	0.98, 0.02
6MWD (B [95% CI])	0.01 [0.004, 0.02]	0.02 [-0.01, 0.06]	**0.09 [0.05, 0.13]**	0.02 [-0.01, 0.06]	0.02 [-0.02, 0.05]
6MWD (β, *p*)	0.46, 0.005	0.21, 0.229	**0.60, <0.001**	0.24, 0.173	0.17, 0.327

Note: Associations that survive FDR correction are highlighted in bold; * - indicates that values are uncorrected standardized scores; †- indicates that values were winsorized; 30s Chair Stand – 30-second chair stand; TUG – timed up and go; 6MWD – 6-minute walk distance; MoCA – Montreal Cognitive Assessment; LSWM – List Sort Working Memory; PSM – Picture Sequence Memory; DCCS – Dimensional Change Card Sort
